# The Algal Chloroplast as a Testbed for Synthetic Biology Designs Aimed at Radically Rewiring Plant Metabolism

**DOI:** 10.3389/fpls.2021.708370

**Published:** 2021-09-24

**Authors:** Harry O. Jackson, Henry N. Taunt, Pawel M. Mordaka, Alison G. Smith, Saul Purton

**Affiliations:** ^1^Department of Structural and Molecular Biology, University College London, London, United Kingdom; ^2^Department of Plant Sciences, University of Cambridge, Cambridge, United Kingdom

**Keywords:** crop improvement, chloroplast, synthetic biology, transplastomics, *Chlamydomonas reinhardtii*

## Abstract

Sustainable and economically viable support for an ever-increasing global population requires a paradigm shift in agricultural productivity, including the application of biotechnology to generate future crop plants. Current genetic engineering approaches aimed at enhancing the photosynthetic efficiency or composition of the harvested tissues involve relatively simple manipulations of endogenous metabolism. However, radical rewiring of central metabolism using new-to-nature pathways, so-called “synthetic metabolism”, may be needed to really bring about significant step changes. In many cases, this will require re-programming the metabolism of the chloroplast, or other plastids in non-green tissues, through a combination of chloroplast and nuclear engineering. However, current technologies for sophisticated chloroplast engineering (“transplastomics”) of plants are limited to just a handful of species. Moreover, the testing of metabolic rewiring in the chloroplast of plant models is often impractical given their obligate phototrophy, the extended time needed to create stable non-chimeric transplastomic lines, and the technical challenges associated with regeneration of whole plants. In contrast, the unicellular green alga, *Chlamydomonas reinhardtii* is a facultative heterotroph that allows for extensive modification of chloroplast function, including non-photosynthetic designs. Moreover, chloroplast engineering in *C. reinhardtii* is facile, with the ability to generate novel lines in a matter of weeks, and a well-defined molecular toolbox allows for rapid iterations of the “Design-Build-Test-Learn” (DBTL) cycle of modern synthetic biology approaches. The recent development of combinatorial DNA assembly pipelines for designing and building transgene clusters, simple methods for marker-free delivery of these clusters into the chloroplast genome, and the pre-existing wealth of knowledge regarding chloroplast gene expression and regulation in *C. reinhardtii* further adds to the versatility of transplastomics using this organism. Herein, we review the inherent advantages of the algal chloroplast as a simple and tractable testbed for metabolic engineering designs, which could then be implemented in higher plants.

## Introduction

The latter half of the 20th century witnessed what is widely referred to as the “Green Revolution”. By implementing agricultural technologies such as novel high-yielding varieties of cereal crops in combination with the adoption of synthetic fertilisers, new crop protection technologies, and intensive irrigation regimes, it is estimated that global food production tripled between the 1960's and early 2000's (Pingali, 2012). However, the returns from such technologies are plateauing for all major crops, particularly for the cereals (Grassini et al., 2013), and it is recognised that more radical changes to the phenotypes of crop species will be needed in order to significantly increase their productivity and improve their nutritional content. Changes that are essential to meet the challenges of dramatic climatic changes and an ever-increasing global population (Bailey-Serres et al., 2019). Carefully managed commercial production of transgenic crops with resistance to herbicides (Duke, 2015) and insect pests (Tabashnik et al., 2013) has already become a reality and has shown promise for future undertakings. One can imagine further desirable improvements, such as tolerance to weather extremes (Bailey-Serres et al., 2019), more efficient light harvesting and energy conversion through manipulations of the photosynthetic apparatus (Perera-Castro and Flexas, 2020), “upgrading” the carbon-fixation pathway (Simkin et al., 2015, 2017; Ding et al., 2016) and even the elimination of the ubiquitous but inefficient Rubisco enzyme (Sharwood, 2017; Cummins et al., 2018). Further radical engineering might include the introduction of nitrogen fixation into the plant tissue (Liu et al., 2018) or new anabolic pathways for key nutrients currently lacking in plants, such as vitamin B_12_ (Smith et al., 2007) and long-chain polyunsaturated fatty acids (Venegas-Calerón et al., 2010). Clearly, realising such goals will require approaches beyond conventional breeding and selection, namely sophisticated genetic engineering tools combined with synthetic biology and systems biology technologies for the major crop species, together with a simple model chassis that can serve as a tractable and malleable testbed for exploring these radical ideas.

**Figure 1:**
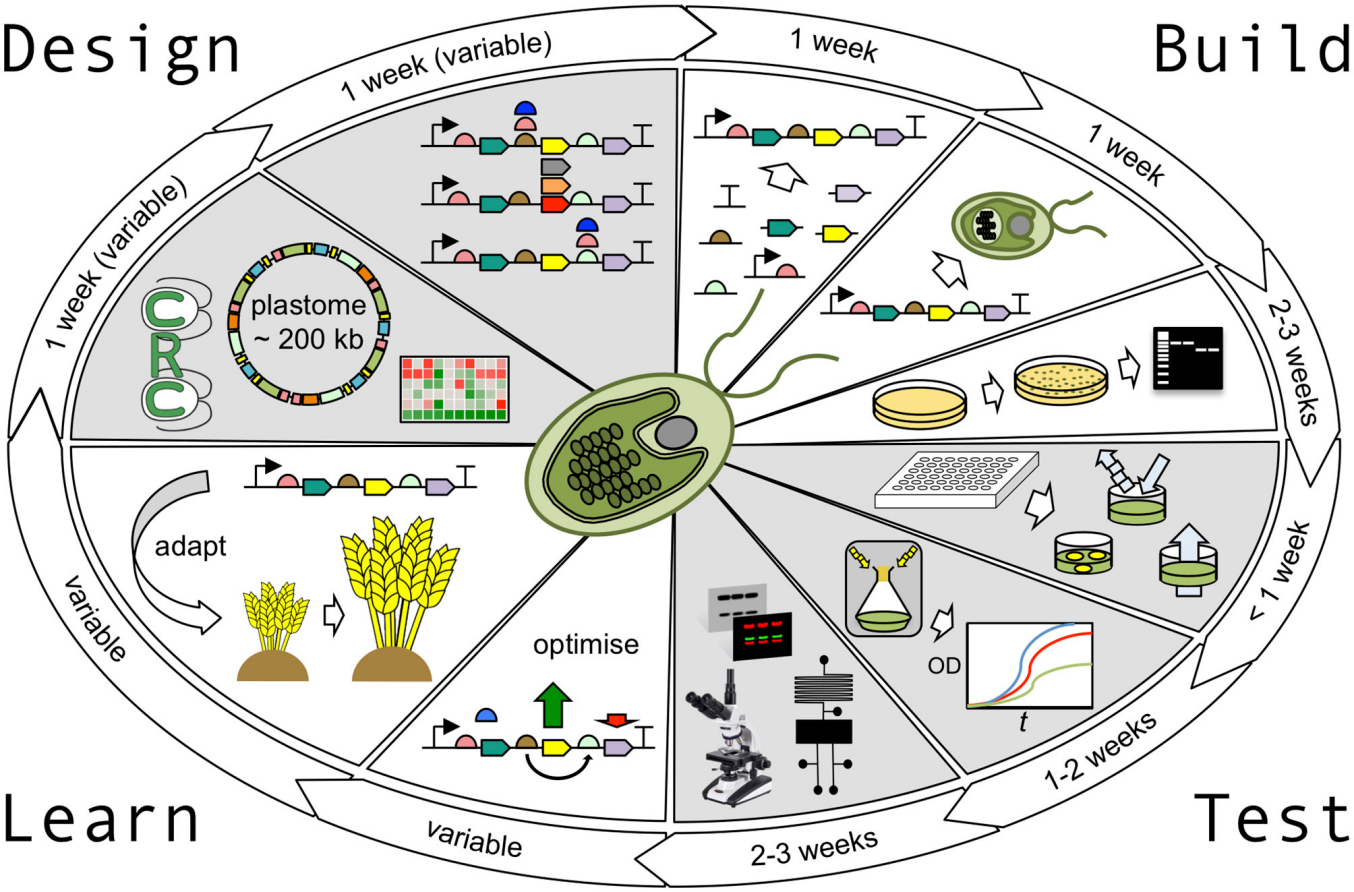
The *Chlamydomonas* plastid testbed. The design stage of the Design-Build-Test-Learn (DBTL) cycle is supported by the availability of accurately detailed genomic and transcriptomic information and a vast library of parental mutant strains, readily available from the *Chlamydomonas* Resource Center. Golden Gate cloning strategies can be used to design parallel builds of multiple construct variants with ease. In the build phase, these constructs can then be assembled in one-pot cloning reactions to generate validated plasmids in less than a week. The chloroplast transformation procedure takes ~1 week, including culturing time, with colonies taking ~1 week to appear and a further ~3 weeks to generate homoplasmic transgenic cell lines. In the test phase, rapid absorbance, fluorescence, and luminescence assays can be carried out in <1 week to screen cell lines before more robust growth analysis (1–2 weeks), and sophisticated physiological analysis is performed (2–3 weeks). Depending on the output of the test phase, designs can either be optimised further or adapted for use in higher plants. The entire DBTL cycle can be as short as 10–13 weeks.

For many of the desired improvements, the plastid is a key player since it is the site of photosynthesis within green tissue, as well as the site of major metabolic pathways, such as starch, haem, and fatty acid biosynthesis (Neuhaus and Emes, 2000), and a proposed location for nitrogen fixation (Liu et al., 2018). Since plastids contain their own small genome (termed, the plastome) and a genetic system derived from their prokaryotic ancestry (Green, 2011), this represents an attractive target for genetic engineering compared to the nuclear genome. Here, novel enzymes or structural proteins would be synthesised *in situ* in the plastid rather than requiring import from the cytosol, and the physical separation of the plastome from the nuclear genome may allow for more radical modifications, such as genetic recoding for biocontainment of the transgenes (Clark and Maselko, 2020). Genetic engineering of the plastome (transplastomics) is well-established in *Nicotiana tabacum* and is feasible for approximately 20 other plant species (Bock, 2015; Yu et al., 2020), and we are now seeing the development of synthetic biology (SynBio) assembly methods and libraries of validated DNAs that will allow for the rapid design and construction of different transgene assemblies (Occhialini et al., 2019). Ideally, these should be tested via multiple cycles of Design-Build-Test-Learn (DBTL; Figure 1) to explore the engineering space. However, implementation is challenging, not least because the obligate phototrophy of these plants makes manipulations of photosynthetic function problematic, and their macroscopic size and the many months needed to generate mature transgenic plants limit the scale and speed of DBTL cycles (Schindel et al., 2018). In contrast, the unicellular microalga, *Chlamydomonas reinhardtii* (hereafter *Chlamydomonas*) is able to dispense completely with photosynthesis and possesses a single chloroplast that is readily transformable (Boynton et al., 1988; Chen, 1996; Wannathong et al., 2016). Transplastomic lines can be generated in only a few weeks, and the microbial nature of *Chlamydomonas* means it is amenable to high-throughput analysis, either as colonies on agar plates (Shao and Bock, 2008; Ferenczi et al., 2017; Nouemssi et al., 2020), liquid cultures arrayed in microtiter plates (Haire et al., 2018), or as single cells sorted using flow cytometry or microdroplet technology (Pan et al., 2011; Velmurugan et al., 2013; Kim et al., 2017; Yu et al., 2021).

Since the first demonstration of chloroplast transformation of *Chlamydomonas* in 1988 (Boynton et al., 1988), the molecular tools and the know-how for manipulating the plastome have steadily improved, and we are now seeing the emergence of SynBio technologies that allow for the reduction and refactoring of the plastome, the standardised assembly and targeted insertion of multiple transgenes, and the analysis and regulation of these genes (Gimpel et al., 2016; Larrea-Alvarez and Purton, 2020). Importantly, algal transplastomics is now spreading beyond this model species with a flurry of reports of chloroplast transformation in other microalgae, including commercially-important species that are gaining traction as future sources of food and feed (Georgianna et al., 2013; Cui et al., 2014; Gan et al., 2018). If these results can be reproduced and implemented, improving the productivity and nutrition of these species will provide at least a partial solution to feeding the world.

In this review, we discuss the evolution and tractability of the chloroplast genetic system, recent advances in genetic engineering technologies for the algal chloroplast, the emergence of SynBio approaches, and the potential for the application of DBTL in the context of the algal chloroplast. We summarise examples of projects for which there is great potential for the algal chloroplast as a SynBio testbed for the kind of advanced, step-change modifications needed to power the Green Revolution 2.0.

## Evolution and Tractability of the Chloroplast Genetic System

The chloroplasts of plants and algae evolved from a single endosymbiotic event ~1.5 billion years ago when a cyanobacterial ancestor was engulfed by a heterotrophic eukaryote (Green, 2011). The evolution of the chloroplast involved multiple gene losses and extensive transfer of genes to the host-cell nucleus. Consequently, the plastome has undergone a dramatic reduction in size and complexity compared to the original cyanobacterial genome, with ~90% of chloroplast proteins now being encoded by the nucleus and imported into the organelle. Most of the hundred-or-so genes that have been retained on the plastome encode core components of the photosynthetic apparatus or the transcription–translation machinery of the organelle. The plastid can therefore be viewed as a naturally evolved minimal cell, containing a streamlined genome and genetic system that retains many prokaryotic features (Scharff and Bock, 2014). The nucleus controls much of the genetic function of the chloroplast, encoding many of the ribosomal proteins and other housekeeping components, as well as specificity factors for RNA transcription, processing, stability, and translation. At the same time, retrograde signalling from the organelle regulates nucleus-encoded photosynthetic genes in response to the physiological status of the chloroplast (Chan et al., 2016).

Whilst targeting of foreign genes into the plastome has been reported for various higher plants and algae, it is most established for tobacco and *Chlamydomonas* (Purton et al., 2013; Yu et al., 2020). Transformation of the *Chlamydomonas* chloroplast genome was first demonstrated over 30 years ago, with DNA delivered into the organelle by the bombardment of lawn of cells with DNA-coated microparticles (Boynton et al., 1988). This “biolistics” method is highly effective with initial transformant colonies recovered after ~7 days (Purton, 2007) and has been routinely adopted in many labs around the world. Alternatively, a simpler, although less efficient, method for the transformation of the *Chlamydomonas* chloroplast involves vortexing a cell suspension with glass beads and DNA (Larrea-Alvarez et al., 2021). DNA insertion into the plastome occurs exclusively *via* homologous recombination, allowing for precise and predictable integration of transgenes (Bock, 2015). However, there are between ~40 and ~100 copies of the plastome in each chloroplast, depending on the physiological state of the cell (Lau et al., 2000), and only a few copies initially acquire the foreign DNA (so-called heteroplasmy). It is, therefore, necessary to drive the transformant lines to homoplasmy by single colony isolation on selective media; this process takes a further 2–3 weeks for *Chlamydomonas*, whereas for plants that possess multiple chloroplasts per cell (so as many as 10,000 plastome copies per cell) this can require multiple rounds of plant regeneration from leaf tissue and can take several months (Bock, 2015).

**Figure 2:**
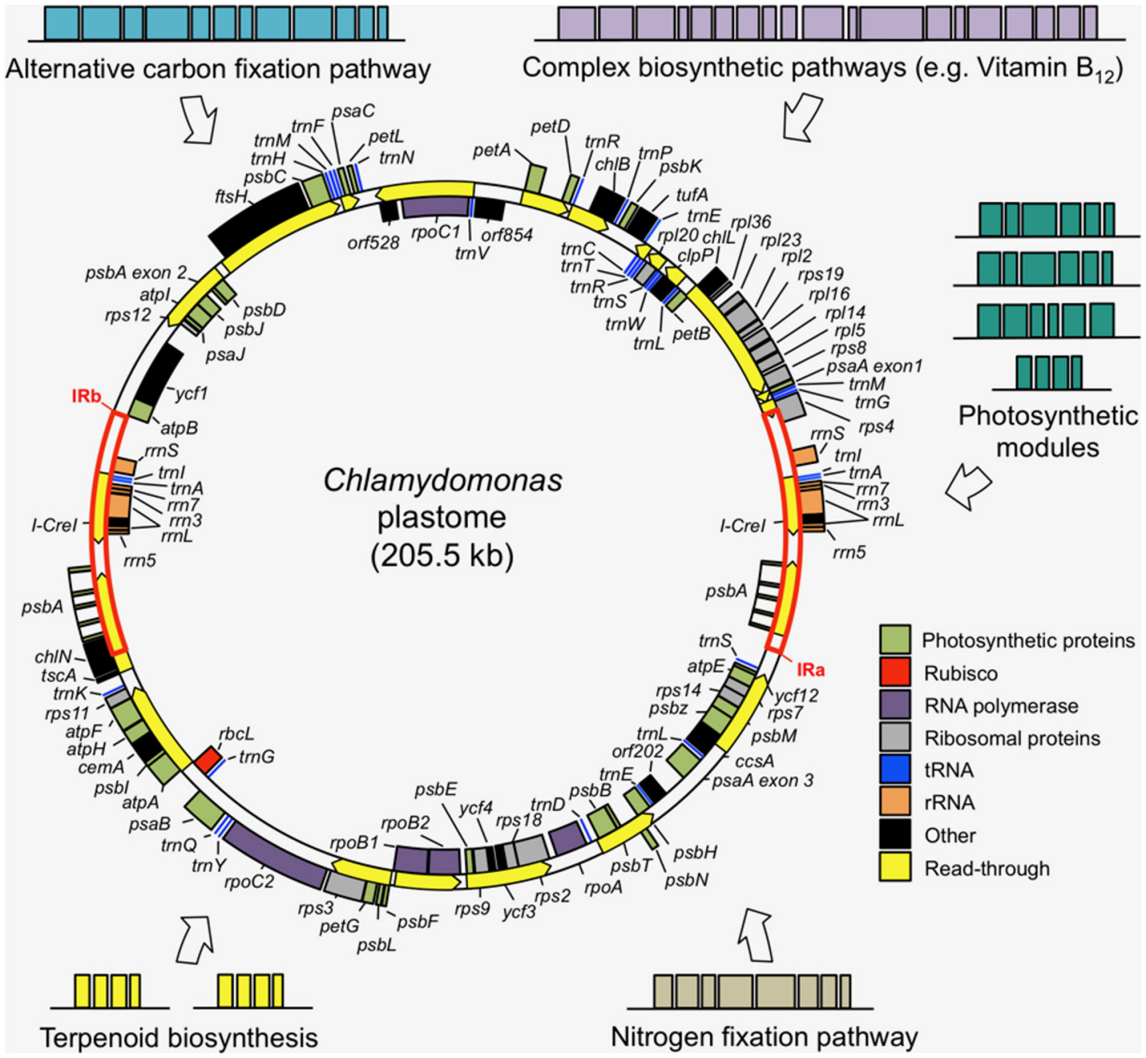
Examples of transgene modules that could be added to the *Chlamydomonas* plastome to introduce novel metabolisms into the chloroplast. The wild-type plastome is composed of two single copy regions separated by two inverted repeats (IRa and IRb), with most genes encoding key subunits of the photosynthetic complexes and the transcription and translation machinery. The simplicity and tractability of the plastome make it a well-suited genetic system for complex metabolic engineering efforts. The plastome map was produced using the Cpv4 genomic data (Gallaher et al., 2018) with transcriptional read-through annotations (yellow arrows) from Cavaiuolo et al. (2017).

The DNA sequence of the 205 kb circular plastome of *Chlamydomonas* was first assembled in 2002, revealing a typical chloroplast DNA structure of two single-copy regions separated by two large inverted repeats (Maul et al., 2002). The presence of many short-dispersed repeats (SDRs) was observed as a major contributor to the high repetitive sequence content of the plastome (~20%). An improved plastome sequence, derived from a single strain (CC-503) was produced in 2009 (Smith and Lee, 2009), before a *de novo* sequence of the complete plastome was generated by shotgun sequencing in 2018 and complemented with RNA-Seq-guided gene annotations, amending several discrepancies in previous versions (Gallaher et al., 2018). The *Chlamydomonas* plastome encodes 108 genes (including duplicates) (Gallaher et al., 2018): 8 rRNA genes, 29 tRNAs, and 71 protein-coding genes (Figure 2), a typical gene content for chloroplast genomes (Green, 2011).

Gene expression in the *Chlamydomonas* chloroplast is simpler than that in higher plant plastids. Transcription involves a single eubacterial type of RNA polymerase and promoter recognition mediated by a single sigma^70^-like factor (Smith and Purton, 2002). This contrasts with plant plastids that possess two or more RNA polymerases and multiple sigma factors with distinct promoter preferences (Puthiyaveetil et al., 2021). All of the transcription start sites on the *Chlamydomonas* plastome have recently been mapped, together with most of the RNA processing sites (Cavaiuolo et al., 2017). Only three gene transcripts (those for *psaA, psbA*, and *rrnL*) undergo RNA splicing, and unlike in higher plants (Rodrigues et al., 2017), there is no RNA editing. Post-transcriptional steps of transcript processing, stabilisation, and translation initiation are mediated by nuclear-encoded factors, most of which are pentatricopeptide repeat (PPR), tetratricopeptide repeat (TPR), or octatricopeptide repeat (OPR) proteins (Gorchs-Rovira and Smith, 2019). Many of the *cis* elements on chloroplast transcripts that are the binding targets for these factors have been mapped (Cavaiuolo et al., 2017), and an increasing number of the factors themselves have been characterised in *Chlamydomonas*, including identification of their target genes (Johnson et al., 2010; Jalal et al., 2015; Marx et al., 2015; Cline et al., 2017; Viola et al., 2019; Ozawa et al., 2020).

The study of photosynthesis-related genes in *Chlamydomonas* has been greatly aided by its facultative heterotrophy. When provided with acetate as a source of fixed carbon, *Chlamydomonas* is able to dispense completely with photosynthesis and grow as a heterotroph. This key feature has enabled researchers not only to knock out most of the 37 photosynthetic genes, thus experimentally validating their dispensability (Rochaix, 2002) but also to generate mutations in hundreds of photosynthesis genes carried by the haploid nuclear genome. The functions of several *ycf* genes (h*y*pothetical chloroplast open reading *f* rame), conserved in the plastomes of algae and in higher and lower plants, have also been elucidated through the analysis of *Chlamydomonas* knockout mutants (Boudreau et al., 1997). As a consequence, a vast array of mutants have been isolated that are defective in key photosynthetic processes, such as light capture, electron transfer, carbon fixation, and pigment biosynthesis (Harris, 2009; Dent et al., 2015). The availability of such mutants, together with a simple sexual cycle, and the ability to genetically engineer both the nuclear and chloroplast genomes has resulted in *Chlamydomonas* becoming an important reference organism for molecular-genetic research into photosynthesis and chloroplast biology (Salomé and Merchant, 2019).

## Emerging SynBio Tools for Engineering the *Chlamydomonas* Plastome

Early interest in *Chlamydomonas* was centred on basic research, but in the last two decades, interest has grown in the use of this algal species as a production platform. This has mainly focused on the light-driven synthesis of high-value therapeutic proteins and metabolites (Gimpel et al., 2015; Dyo and Purton, 2018; Taunt et al., 2018; Lauersen, 2019); however, with the increasing availability of SynBio tools, such as interchangeable parts and high throughput assembly tools, the goals of such applied projects have become more ambitious. This section describes the progress made in the development of SynBio in the algal chloroplast.

### Gene Regulation Tools

Successful therapeutic protein production platforms are heavily reliant upon the ability to achieve high levels of transgene expression, and even for metabolic engineering sufficient levels of heterologous enzyme expression are required to ensure adequate substrate capture from endogenous metabolism and sufficient production rates for downstream steps. Moreover, the ability to precisely regulate gene expression to fine-tune metabolic pathways is important for optimal yields. For high constitutive expression of transgenes, endogenous chloroplast 5′-untranslated regions (5′-UTRs) of photosynthetic genes, such as that of the *psbA* and *psaA* genes, have typically been used in combination with the strong 16S ribosomal RNA promoter (Rasala et al., 2011; Bertalan et al., 2015). The expression strength of these endogenous 5′-UTR sequences is often limited by negative feedback regulation that would prevent the over-accumulation of photosynthetic subunits in the native context of the corresponding genes (Choquet and Wollman, 2002). Consequently, the 5′-UTR is often the limiting factor for transgene expression (Coragliotti et al., 2011). A promising strategy to mitigate this is the use of synthetic 5′-UTR variants that are not subject to these feedback mechanisms. Such variants have already been generated and used to improve the transgene expression (Specht and Mayfield, 2013).

Enabling advancements have recently been made in the development of tools for regulating gene expression in the *Chlamydomonas* chloroplast. One such system uses a modified version of a synthetic tRNA gene (*trnW*_*UCA*_*)*, which encodes a temperature-sensitive tRNA that allows for the translation of an unused stop codon (TGA) in the *Chlamydomonas* chloroplast as a tryptophan codon. The translation of transgenes that have been modified to contain one or more internal TGA codons can then be tuned by shifting the culture temperature from 35°C (translation stalls at the internal stop codons) to 18°C (translational read-through occurs). The induction behaviour can be further tailored by changing the number of TGA codons incorporated within the transgene (Young and Purton, 2016, 2018).

Many nuclear-encoded factors have been shown to regulate the expression of specific chloroplast genes at the posttranscriptional level, often by binding to target sites located in the 5′-UTR of the mRNA (Barkan and Goldschmidt-Clermont, 2000). Knowledge of such factors has been exploited to establish chloroplast repressible systems (Rochaix et al., 2014). For example, the nuclear-encoded NAC2 protein stabilises the *psbD* mRNA encoding the D2 protein of photosystem II by binding to the 5′-UTR of the transcript (Nickelsen et al., 1994). Fusion of the *NAC2* gene to the inducible promoter of the *CYC6* gene, which is tightly repressed when copper is present in the growth medium and strongly expressed under copper deprivation or in the presence of nickel (Quinn et al., 2002), and introduction of this chimeric gene into the nuclear genome of a *nac2* mutant, has been shown to permit the regulation of the chloroplast, *psbD* 5′-UTR by changing the copper/nickel content of the growth medium (Surzycki et al., 2007; Rochaix et al., 2014). This repressible system can be applied to any chloroplast gene of interest by switching its 5′-UTR to that of *psbD*, with the option of preserving photosystem II expression and assembly under repression conditions by introducing a version of the *psbD* gene under the control of a 5′-UTR that is not responsive to NAC2 (Surzycki et al., 2007). Similarly, thiamine-based nuclear regulation of chloroplast transgenes has been established by the use of a TPP-responsive riboswitch in the *THI4* gene (Croft et al., 2007). Inclusion of the thiamine riboswitch in the NAC2 construct allows for its expression to be repressed in the presence of thiamine (Ramundo and Rochaix, 2015). Another recently established nuclear inducible chloroplast regulation system uses the C-terminus of the nuclear-encoded factor, TDA1 (cTDA1) that regulates the stability and translation of *atpA* transcripts through interactions with its 5′-UTR. The cTDA1, under the control of the chimeric promoter, *HSP70A-RBCS2*, was introduced into the nucleus of a chloroplast transformant expressing green fluorescent protein (GFP) under the control of the *atpA* 5′-UTR, enabling the control of GFP levels by light and heat-shock treatments (Carrera-Pacheco et al., 2020).

### Multigenic Expression

To date, there have been reports of over 100 different foreign proteins produced in the *Chlamydomonas* chloroplast (Larrea-Alvarez and Purton, 2020) Whilst these studies have typically involved the introduction of a single transgene and selection marker into the plastome, reports of multigenic genetic engineering approaches involving up to six transgenes are now appearing (Gimpel et al., 2016; Macedo-Osorio et al., 2018; Larrea-Alvarez and Purton, 2020). Multiple genes can be integrated as discrete transcriptional units, each under the control of a dedicated promoter, and 5′- and 3′-UTRs. However, the use of the same *cis* element in more than one transcription unit needs to be avoided to prevent unwanted recombination events between direct repeats leading to transgene instability (Gimpel et al., 2016; Larrea-Alvarez and Purton, 2020). An alternative strategy to creating separate transcriptional units is to express multiple transgenes as a single synthetic operon (Bock, 2013; Macedo-Osorio et al., 2018; Hsu et al., 2019). Many endogenous genes in the chloroplast are co-expressed from a single promoter as polycistronic transcripts separated by “intercistronic expression elements” (IEEs). The IEEs facilitate the processing of the primary transcript to yield translatable monocistronic mRNAs (Bock, 2013). These IEEs are useful tools for stacking genes in synthetic operons for chloroplast metabolic engineering in both *Chlamydomonas* and tobacco. For example, three key genes involved in the tocochromanol (Vitamin E) pathway were assembled into a synthetic operon in tobacco, resulting in a 10-fold increase in tocochromanol accumulation compared to the expression without the use of the IEEs (Lu et al., 2013).

### The Arrival of Standardised Modular Cloning Platforms, Such as Golden Gate

The rate at which complex DNA constructs can be designed and assembled has historically been a bottleneck for basic molecular research. However, the implementation of rapid and standardised assembly methods, such as Golden Gate, Gibson Assembly, and ligase cycling reaction (Casini et al., 2015) have led to a paradigm shift in the capabilities for genetic engineering, facilitating the application of the SynBio DBTL approach. Golden Gate methods are particularly well-suited for the hierarchical, high-throughput assembly of the multiple constructs needed to explore the design space. Briefly, these technologies use a single Type IIS restriction enzyme for the cutting and joining of all DNA parts, thereby eliminating the context-specific nature that is true of traditional cloning methods. Golden Gate relies on a specific and universal “syntax” (i.e., the sequence of a single-stranded overhang generated by digestion with the type IIS enzyme) for each fundamental DNA “part” or “level 0” (these include coding sequences, promoters, UTRs, IEEs, terminators, etc.) (Weber et al., 2011). Individual transcriptional units (“level 1”) are then assembled from the chosen level 0 parts in a single digestion/ligation reaction with the syntax ensuring the joining of parts in the correct order and orientation within the designed construct. The constructs of Level 1 are then similarly assembled into higher level constructs containing one or more transcription units, a selectable marker, and other elements required in the final transformation plasmid. These approaches have given rise to modular cloning (MoClo) kits for nuclear transformation of a range of organisms including yeast (Lee et al., 2015), plants (Noor-Mohammadi et al., 2012; Engler et al., 2014), and animal cells (Vasudevan et al., 2019), and are now being employed for the *Chlamydomonas* nucleus (Crozet et al., 2018) and chloroplast (Noor-Mohammadi et al., 2012; Oey et al., 2014; Bertalan et al., 2015). A recent adaptation of the Golden Gate method, known as “Start-Stop Assembly,” has been developed for bacteria (Taylor et al., 2019), and is also particularly applicable to chloroplast engineering. Central to the method is a 3-base syntax, with the DNA joinings at either end of the coding sequence part corresponding to the start and stop codons (ATG and TAA, respectively), which allows for completely scar-free assembly of coding sequences into the expression units. The further development and adoption of this technology will significantly advance chloroplast SynBio research by reducing the time and effort needed to complete each DBTL cycle, increasing the number of different plasmid designs that can be assembled in parallel and fed into each cycle, and by reducing the duplication of efforts and inconsistencies between research groups with a library of standardised and validated parts that can be readily shared.

### Whole Plastome Engineering

The design of a highly reduced synthetic genome, or “minimal genome” in which all non-essential genes are removed, and its successful transplantation into a recipient cell or organelle is widely regarded as one of the next great challenges for SynBio (Lachance et al., 2019). Such systems not only provide a bottom-up approach to exploring fundamental molecular processes within a simplified cell or organelle but also provide a naïve chassis onto which can be bolted new desirable traits or a drastically restructured metabolism. In the case of a minimal synthetic plastome (Scharff and Bock, 2014), this could allow for the remodelling of large elements of the photosynthetic apparatus without interference from the native system, for example, introducing entire photosystems from other organisms and evaluating their performance. The *Chlamydomonas* plastome is particularly well-suited for such an undertaking given the non-essential nature of all the photosynthetic genes, the detailed knowledge of its transcriptome, and the SynBio resources available for subsequent genetic engineering of the minimal plastome. In a first exploration of this, O'Neill et al. (2012) assembled a variant of the entire *Chlamydomonas* plastome in yeast and introduced it into the chloroplast. However, this resulted in the formation of chimeric forms of the plastome due to unwanted DNA recombination between the native and introduced versions. This illustrates a major challenge that must be overcome for a clean plastome transplantation: namely, the need to develop a method for selective elimination of the native DNA either prior to or immediately following transformation. Nevertheless, a simple *in silico* analysis of all the non-essential regions of the *Chlamydomonas* plastome (i.e., all the photosynthetic genes, one copy of the large inverted repeat, the short-dispersed repeats, and the *rrnL* intron) leads to a design that is ~96 kb in size and possesses just 57 RNA and protein genes. This minimal synthetic plastome could be easily assembled and propagated in yeast prior to transplantation into the algal chloroplast. In addition to minimisation, another improvement that could be made to generate a powerful SynBio chassis strain is the re-factoring of genes into functionally defined clusters on the synthetic plastome, as has been done for six genes encoding Photosystem II subunits (Gimpel et al., 2016). Extension of this more broadly would allow for a modular approach to plastome engineering in which whole sets of genes can be easily replaced or additional gene modules bolted on during the design/build stage without requiring *de novo* synthesis of the whole plastome.

## Design-Build-Test-Learn Strategies

The Design-Build-Test-Learn cycle (Figure 1) is a systematic and iterative approach in SynBio and strain development that is being widely adopted in metabolic engineering to obtain a design that satisfies a given specification (Pouvreau et al., 2018; Opgenorth et al., 2019). The critical feature is that rather than one design, researchers explore parameter space to find an optimal engineered solution to the problem. With the currently available genomic/transcriptomic resources for *Chlamydomonas* and the efficient DNA assembly methods, the Design and Build stages of the DBTL cycle can be performed in a matter of days. Indeed, high-throughput parallel assemblies of multiple DNA constructs for chloroplast engineering are possible in less than a week once all parts are available, either as pre-existing parts within a library or by the generation of new parts through PCR amplification of genomic DNA/cDNA or gene synthesis (Noor-Mohammadi et al., 2012; Oey et al., 2014; Bertalan et al., 2015; Crozet et al., 2018; Gallaher et al., 2018; Occhialini et al., 2019).

In order to test the efficacy of engineering efforts, precise techniques for monitoring basic physiology, protein expression levels, and genomic stability are of vital importance. Growth analysis of *Chlamydomonas* can be performed easily and robustly at a range of scales, either in microplate format (Haire et al., 2018), at flask-scale using lab-scale photobioreactors that offer precision control of environmental conditions, or in mid-scale commercial bioreactors (Changko et al., 2020). Amongst the DNA parts available are an array of fluorescent and luminescent reporter genes for studying nuclear and chloroplast gene expression *in vivo* in *Chlamydomonas* (Rasala et al., 2013; Lauersen et al., 2015; Esland et al., 2018). These reporters provide a rapid and high-throughput method to assess gene expression and can be made quantitative through the use of recombinant standards. Alternatively, quantitative measurements of recombinant proteins can be achieved *via* Western blot by incorporating an epitope tag into the design of the protein (Stoffels et al., 2017; Larrea-Alvarez and Purton, 2020).

Imaging techniques for *Chlamydomonas* have also undergone significant improvement in recent years and will serve as powerful diagnostics tools for chloroplast engineering. Confocal microscopy is routinely used to visualise whole cell architecture *via* bright-field, or for fluorescence analysis and visualisation of endogenous or recombinant fluorescent proteins (Rasala et al., 2013). Live imaging of nucleoids (the DNA–protein conglomerates within chloroplasts that contain multiple copies of the plastome) within single cells has been performed using strains expressing a YFP-tagged histone-like DNA binding protein, as was deployed in conjunction with microfluidic technology to reveal the dynamic nature of nucleoid networks over the course of cellular division (Kamimura et al., 2018). *In situ* cryo-electron tomography has now made it possible to visualise individual Photosystem I and Photosystem II (PSI & PSII) complexes within the thylakoid membrane with stunning detail, laying the foundation for the use of this technology to observe photosynthetic regulation at the level of single-protein complexes (Wietrzynski et al., 2020).

Together, these technologies for engineering and analysing the *Chlamydomonas* plastid enable DBTL to be carried out in very short order, in turn accelerating the rate of progress of basic and applied SynBio studies. This knowledge could then be adapted for more complex multicellular systems, such as higher plants, to realise the ambitious metabolic redesign goals for these species on a more relevant time scale.

## Priority Projects for the Testbed

There are many potential trait adjustments to plant crops that could help them cope with the increasing production demands and potentially extreme environmental challenges of the future. Of the many SynBio projects that could be envisaged, some priority projects that lend themselves well to the algal chloroplast testbed are briefly discussed in this section and illustrated in Figure 2.

### Improving Photosynthesis

The microalgal chloroplast has been used extensively for the study of photosynthesis and more recently as a target for improving the efficiency of photosynthesis and the overall productivity of light-driven production systems. Among the proposed strategies that are applicable to higher plants is the expansion of the spectral band used for photosynthesis by the introduction of additional light-harvesting pigments (Blankenship and Chen, 2013; Trinugroho et al., 2020), enhancing photoprotective mechanisms (Leister, 2019), and even the introduction of recombinant photosystems (Gimpel et al., 2016).

The high degree of evolutionary constraint on the essential photosynthetic complexes, PSI & PSII and the cytochrome *b*_6_*f* complex, has resulted in the so-called “frozen metabolic state” (Gimpel et al., 2016). Modification to these complexes is made challenging by the strong conservation of the structural components and the need for simultaneous modification of multiple protein subunits, often encoded in both the nucleus and the chloroplast genomes (Leister, 2019). Re-factoring of the six-gene PSII core in the chloroplast of *Chlamydomonas* illustrated that there were additional limiting interactions beyond the core subunits that reduced the overall quantum yield of recombinant variants (Gimpel et al., 2016). The use of ‘synthetic photosynthetic modules’ that contain a sufficient number of the necessary proteins, genetic elements, and auxiliary factors for the proper functioning of a given complex has thus been highlighted as an important consideration for these SynBio approaches (Leister, 2019).

An alternative to engineering the highly intricate photosynthetic machinery is to introduce a heterologous electron sink that can harvest the reducing power from photosynthesis and use it to power unrelated metabolic processes (Mellor et al., 2017). This concept holds promise as it is not reliant on modifying the photosynthetic machinery but rather harvesting the excess reducing power that would otherwise be dissipated to prevent photodamage. Pioneering studies in cyanobacteria and plant plastids have shown that reducing equivalents from PSI can be redirected to cytochrome P450 enzymes and, due to the extreme versatility of this enzyme family, coupled to a wide variety of metabolic processes (Lassen et al., 2014a,b; Berepiki et al., 2016; Nielsen et al., 2016). The chloroplast of *Chlamydomonas* would be an ideal platform to develop these strategies due to the ease and speed of genetic engineering as well as the ability to grow the cell mixotrophically in the presence of acetate, thus enabling more reducing power to be harvested from photosynthesis without perturbing cell fitness and growth. A gene (*CYP79A1*) encoding a cytochrome P450 from *Sorghum bicolor* has already been introduced into the *Chlamydomonas* chloroplast, resulting in the stable expression of the P450 and its targeting to the chloroplast membrane. The heterologous P450 was shown to catalyse the first reaction (conversion of tyrosine to p-hydroxyphenylacetaldoxime) of the biosynthesis pathway of dhurrin, a plant defence compound (Gangl et al., 2015). This work provides a proof of concept for the light-driven production of complex plant metabolites in the algal chloroplast using cytochrome P450s.

### Carbon Fixation

Improving the efficiency with which plant crops convert CO_2_ into biomass will be key to meeting the agricultural yield requirements of an ever-growing global population. Accordingly, improving the rate of carbon fixation in plants has been a target for metabolic engineering (Kubis and Bar-Even, 2019). Proposed strategies for boosting carbon fixation include engineering a more efficient Rubisco (Loganathan et al., 2016; Sharwood, 2017), optimisation of the expression levels of Calvin cycle enzymes (Simkin et al., 2015), design and integration of alternative computationally derived synthetic pathways for carbon fixation (Siegel et al., 2015), and introduction of algal carbon-concentrating mechanisms (Long et al., 2016; Rae et al., 2017; Mackinder, 2018). Critical to the success of such efforts is the ability to regulate and tune the expression of genes of a synthetic pathway within the complex biological context of the host organism (Kubis and Bar-Even, 2019). As such, the algal plastid could provide a suitable platform to carry out basic research given the on-going development of engineering tools for the regulation of gene expression and the relative simplicity of the plastid genetic system, as discussed in the previous sections.

For example, a long-standing goal in carbon assimilation research has been to address the limitations of the carbon-fixation enzyme, Rubisco (Sharwood, 2017). In theory, it should be possible to use a rational design approach to improve the rate of catalysis of the enzyme, and its relative affinity for CO_2_ over the competing substrate O_2_. This could then pave the way for plant varieties in which the abundance of Rubisco in green tissue is significantly reduced whilst rates of carbon-fixation are increased. Early efforts to produce a functional recombinant Rubisco in *Escherichia coli* for such design studies were unsuccessful because of the lack of native chaperones required for the folding and assembling of the large (L) and small (S) subunits into the L_8_S_8_ structure. More recently, this issue has been addressed through the co-expression in *E. coli* of multiple chloroplast chaperones from *Arabidopsis* together with the large and small subunits (Aigner et al., 2017). Using this *E. coli* system to explore the design space now allows for the discovery of superior L- and S-subunit variants that can then be tested by introduction into plant models. However, replacement of the endogenous plant genes is complicated by their separate genomic locations, with the L-subunit gene (*rbcL*) located in the plastome and the S-subunit gene family (*RBCS*) in the nuclear genome. Researchers have sought to address this using RNA interference-mediated gene silencing to generate master-lines of *Nicotiana tabacum* in which the nuclear *RBCS* gene is suppressed, enabling the production of Rubisco variants by the transformation of the chloroplast with an *rbcL-rbcS* operon (Martin-Avila et al., 2020). Placing the S-subunit gene in the plastome simplifies comparative studies since both genes can be targeted to a specific locus in a single transformation step. However, the use of *N. tabacum* to test the initial designs is not ideal, given its obligate phototrophy and the limitations for rapid DBTL cycling. *Chlamydomonas* represents an attractive intermediate model since a nuclear mutant is already available in which both members of the *RBCS* gene family are deleted (Khrebtukova and Spreitzer, 1996), and deletion of *rbcL* in the plastome is straightforward (Newman et al., 1991). A double mutant could therefore serve as the recipient for chloroplast transformation using an *rbcS-rbcL* operon. This would allow a rapid assessment of the findings from the *E. coli* studies whereby different combinations of protein engineering within the L- and S-subunits are screened: initially for function by direct selection for restored phototropic growth, and then through biochemical analysis of Rubisco accumulation and catalytic activity. The best designs could then be carried forward for testing in a higher plant such as *N. tabacum*.

### Nitrogen Fixation

Whilst plants are capable of assimilating carbon directly from the atmosphere through photosynthesis, they are not able to directly fix atmospheric nitrogen. Plants are therefore reliant on diazotrophic bacteria and archaea that possess the nitrogen-fixing enzyme, nitrogenase, and are thus able to supply bioavailable forms of this essential element. The Green Revolution was driven in large part by the development of an alternative, chemical method for fixing nitrogen (the Haber–Bosch process) allowing for the production of huge quantities of cheap agricultural fertilisers (Rogers and Oldroyd, 2014). However, there is increasing recognition of the deleterious environmental consequences of our heavy reliance on these chemical fertilisers. These consequences include the consumption of vast amounts of fossil fuels and associated release of CO_2_ during fertiliser production, the release into the atmosphere of large amounts of nitrous oxides following application on the field, and the eutrophication of aquatic ecosystems owing to run-off of excess dissolved nitrates (Sutton et al., 2011). This has incentivised several avenues of research seeking to introduce the capacity for biological nitrogen fixation into crop species (Rosenblueth et al., 2018). One approach is the genetic engineering of cereal crops to improve their association with nitrogen-fixing bacteria that colonise plant roots, with the ultimate goal of reproducing the bacteria-containing root nodules of legumes in the engineered plants (Rogers and Oldroyd, 2014). An alternative approach would be the introduction of the bacterial nitrogenase enzyme into the cells of crop plants such that they are able to directly fix atmospheric nitrogen (Hardy and Havelka, 1975; Rosenblueth et al., 2018). Attempts to engineer plant genomes with the bacterial *nif* genes required for nitrogenase biosynthesis are made challenging by the complexity of the biosynthesis pathway, with as many as 16 gene products required for the functional assembly of the molybdenum-containing metalloenzyme (Curatti and Rubio, 2014). Furthermore, the enzyme is highly oxygen-sensitive, and has a high energy demand with 16 ATP molecules required to convert one N_2_ molecule to NH_3_. However, chloroplasts and mitochondria have sufficient energy reserves to carry out nitrogen fixation and have long been discussed as possible subcellular locations for nitrogenase fixation (Merrick and Dixon, 1984; Beatty and Good, 2011). Recent SynBio efforts involve nuclear engineering of yeast and tobacco such that the *nif* gene products are targeted to the mitochondrion or the chloroplast, respectively, show promising results but also highlight the significant technical challenges of producing a functional nitrogenase enzyme in a eukaryotic organelle (López-Torrejón et al., 2016; Eseverri et al., 2020; Xiang et al., 2020). Furthermore, synthesis of a functional nitrogenase in the chloroplast would require separation from the oxygen evolved by photosynthesis, either temporally, by inducing *nif* gene expression only at night, or spatially, by selectively expressing the *nif* genes in non-photosynthetic tissue (Rosenblueth et al., 2018). *Chlamydomonas* lends itself well to such complex design studies since multiple *nif* genes could be expressed directly in the chloroplast, and the oxygen issue can be circumvented either by using a photosynthetic mutant incapable of oxygen evolution or by culturing the cells in the dark. The capabilities and reduced timescale of the DBTL cycle for chloroplast engineering in this alga could contribute significantly to an iterative effort aimed at solving the many design challenges prior to transferring the technology into crop plants.

### Engineering Crop Plants for the Production of Novel Metabolites

Chloroplast metabolic engineering offers many opportunities for exploiting plants as low-cost and scalable factories for the production of pharmaceutical compounds (so-called plant molecular farming: Obembe et al., 2011), or for developing biofortified crop varieties with high levels of beneficial nutrients such as vitamins, antioxidants, essential amino acids, and minerals (Díaz-Gómez et al., 2017). Whilst some pioneering progress has been made in chloroplast metabolic engineering using plant models, such as tobacco and tomato (Fuentes et al., 2018), it is by its very nature a challenging endeavour since the engineering inevitably perturbs the metabolism of the chloroplast. Multiple cycles of DBTL are therefore necessary to tune the metabolic flux to the novel product and minimise the accumulation of undesirable intermediates. Here, we consider two examples where engineering the *Chlamydomonas* chloroplast can serve as a simple testbed for such studies, namely the synthesis of commercially-important terpenoid compounds and biofortification of crops with vitamin B_12_.

Terpenes are complex linear or cyclic hydrocarbons produced by plants and microorganisms, and are synthesised from multiple units of a 5-carbon branched precursor. Terpenoids are then derived from terpenes through the functionalisation of the hydrocarbon skeleton with oxygen-containing groups, such as hydroxyl, ketone, aldehyde, carbonyl, and peroxide groups, giving rise to a huge diversity of natural compounds (Breitmaier, 2006). Many of these have commercial applications including as pharmaceutical and nutraceutical bioactives, fragrances in cosmetics and cleaning agents, flavourings in food and drinks, and natural insecticides. However, the chiral complexity of terpenoids often precludes their chemical synthesis, and whilst they can be derived from natural plant or microbial sources, the levels are typically very low or present only in specialised organs making the extraction expensive (Vavitsas et al., 2018). Transfer of the biosynthetic pathways into platform species is therefore desirable, and much work has focussed on the biosynthesis of medicinal terpenoids in *E. coli* and yeast, including precursors of the anti-malarial drug, artemisinin and the anti-cancer therapeutic, paclitaxel (Ajikumar et al., 2010; Paddon et al., 2013). Although these two microorganisms are highly advanced platforms for metabolic engineering, the use of plants and algae as alternative platforms offers a number of potential advantages when considering terpenoid biosynthesis (Vavitsas et al., 2018). Specifically, photosynthetic organisms naturally produce a wide variety of terpenoids and devote significant metabolic resources to their biosynthesis. The necessary metabolic infrastructure and high flux potential are therefore already established in these hosts, unlike the situation in *E. coli* and yeast. Furthermore, terpenoid biosynthesis in the chloroplast can be directly linked to photosynthesis *via* electron transfer proteins, thereby providing the reductant necessary for the biosynthesis of terpene skeletons and their functionalisation by heterologous cytochrome P450s (Mellor et al., 2017). Once again, *Chlamydomonas* offers an attractive intermediary production platform to optimise production, with the modular characteristics of terpenoid biosynthesis making it well-suited for systems biology and SynBio approaches to plastid and nuclear genome engineering (Wichmann et al., 2020). Indeed, synthesis of heterologous diterpenoid products, such as casbene, taxadiene, and 13R(+) manoyl oxide has already been demonstrated in the chloroplast of *Chlamydomonas* (Lauersen et al., 2018; Mehrshahi et al., 2020). Diterpenoids produced in the organelle are released from the cell and can be easily captured in dodecane culture overlays for analysis by gas chromatography mass spectrometry, providing a powerful platform for the study of phototrophic heterologous terpenoid production (Lauersen, 2019).

Vitamin B_12_ (B_12_, hereafter) is widely known to be the only vitamin that cannot be acquired directly from plant-derived foods since it is synthesised solely by a subset of prokaryotes (Warren et al., 2002). B_12_ is also by far the most chemically complex of the vitamins with an elaborate 63-carbon tetrapyrrole structure. Fortification of foods with B_12_ or production of B_12_ supplements is therefore dependent on industrial fermentation of bacterial species that natively produce the vitamin (Balabanova et al., 2021). Heterologous biosynthesis of B_12_ has also been achieved by engineering the non-producing bacterium, *E. coli*. This was successful despite the genetic complexity of the biosynthetic pathway, with 28 transgenes from five different bacterial species required for *de novo* synthesis in *E. coli* (Fang et al., 2018). The study serves as a primer for the more challenging but more economic strategy of introducing a B_12_ biosynthetic pathway directly into plant plastids to produce biofortified food crops. To this end, the algal plastid could serve as a useful platform to define the minimum number of enzymes required (given that all the enzymes for the early part of the pathway leading to the synthesis of the related tetrapyrroles, haem and chlorophyll are already located in the chloroplast; Oborník and Green, 2005), and to optimise B_12_ yield through iterative testing of different gene sets and expression levels.

## Concluding Remarks

SynBio research will play a vital role in securing a productive and sustainable future for plant-based agriculture. Among the ambitious improvements proposed for plant crops are the introduction of novel nitrogen/carbon fixation pathways to boost overall biomass production, fortification against environmental threats and extreme weather conditions, and the integration of complex multigene pathways for the production of novel bio-products.

Similar to traditional engineering disciplines, the SynBio approach uses abstraction, decoupling, and standardisation to make the design of complex biological systems more manageable and efficient (Boehm and Bock, 2019). This is illustrated in the systematic characterisation of DNA parts as modular units with a predictable function, which can be organised into genetic systems (or levels) with hierarchical complexity that can be optimised through successive rounds of DBTL (Figure 1).

Synbio research in plant species is still in its infancy and is in need of advancements to the available genetic tools and a deeper understanding of the metabolic systems that are being targeted. As is the case for all host organisms, the time requirement associated with generating transgenic crop plants limits the rate at which the SynBio method of DBTL can be carried out. Furthermore, the space requirement for plant growth also limits the number of different design permutations that can be tested in parallel within a single cycle of DBTL. Clearly, the reduced DBTL timescale and greater numerical capacity of microbial platforms offer significant advantages when exploring the genetic engineering landscape using an iterative approach.

Major breakthroughs using microbial model organisms, such as the re-programming of *E. coli* with a novel synthetic genome (Fredens et al., 2019) and the functional integration of complex metabolic pathways into *E. coli* and yeast (e.g., Paddon et al., 2013; Fang et al., 2018) demonstrate the promise of SynBio. We are now seeing the early stages of the application of SynBio in chloroplast biotechnology (Scharff and Bock, 2014; Schindel et al., 2018; Occhialini et al., 2019), thus opening the possibility of similar breakthroughs in higher plants. Chloroplast SynBio research using single-cell models such as *Chlamydomonas* provides a good starting point and attractive testbed for such ambitious metabolic engineering efforts prior to their exportation to higher plants.

However, the limitations and differences inherent in any model must also be appreciated. *Chlamydomonas* is an aquatic single cell adapted to a mixotrophic lifestyle in freshwater and soil where light, oxygen, and nutrient availability are often limited (Sasso et al., 2018). Its environment is therefore significantly different from that of crop plants, and to consider it a “unicellular plant” is naïve, especially given that chlorophyte algae and land plants diverged from a common ancestor over 800 million years ago (Donoghue and Paps, 2020). *Chlamydomonas* possesses only a single chloroplast per cell compared to the 50–100 chloroplasts typically found in a plant mesophyll cell. Furthermore, plants contain different plastid types depending on the tissue, and the plastids are able to undergo differentiation into different types (Choi et al., 2021). In contrast, the algal chloroplast does not undergo differentiation, and indeed is able to form a fully functional chloroplast when grown in complete darkness, unlike angiosperms (Reinbothe et al., 2010). There are also distinct differences in the finer details of chloroplast gene expression. In *Chlamydomonas*, all chloroplast genes are transcribed by a single eubacterial-type RNA polymerase using a single sigma factor, whereas, in plants, the transcription is shared between eubacterial and bacteriophage-type polymerases with the former employing multiple sigma factors (Puthiyaveetil et al., 2021). Optimisation of parameters for the transcription of transgene clusters using the algal model will not, therefore, be directly transferable to the plant chloroplast. Similarly, RNA editing is a feature of plant chloroplasts that is not seen in *Chlamydomonas* (Small et al., 2020), and might need to be taken into account when sharing DNA parts between the two systems. Finally, differences in the control mechanisms underlying post-transcriptional steps (RNA processing, RNA stability, and translation initiation), as well as codon preference and protein stability, are all likely to affect the expected recombinant protein levels when transferring from algal to plant model (Faè et al., 2017). Nonetheless, despite these caveats, the many advantages of engineering the *Chlamydomonas* chloroplast mean that it has much to offer as a simple and tractable testbed for radical re-engineering of crop species for the 21st century.

## Author Contributions

HJ researched and wrote the review with support from HT and PM who provided valuable feedback and contributions to the text. SP and AS supervised the work and provided critical discussion, feedback, and comments on the manuscript. All authors have approved the submitted version.

## Funding

Chloroplast SynBio research of the authors was funded by the grants BB/R016534/1 and BB/R01860X/1 from the Biotechnology and Biological Sciences Research Council of the United Kingdom.

## Conflict of Interest

The authors declare that the research was conducted in the absence of any commercial or financial relationships that could be construed as a potential conflict of interest.

## Publisher's Note

All claims expressed in this article are solely those of the authors and do not necessarily represent those of their affiliated organizations, or those of the publisher, the editors and the reviewers. Any product that may be evaluated in this article, or claim that may be made by its manufacturer, is not guaranteed or endorsed by the publisher.
